# Evaluating Cataract Surgical Rate through Smart Partnership between Ministry of Health, Malaysia and Federal Territory Islamic Religious Council

**DOI:** 10.3390/medicines10010012

**Published:** 2023-01-12

**Authors:** Nor Fariza Ngah, Nor Asiah Muhamad, Roslin Azni Abdul Aziz, Elias Hussein, Mohammad Aziz Salowi, Zabri Kamarudin, Noor Hisham Abdullah, Tahir Aris

**Affiliations:** 1 Institutes for Clinical Research, Ministry of Health, Shah Alam 40170, Malaysia; 2 Department of Ophthalmology, Shah Alam Hospital, Shah Alam 40000, Malaysia; 3 Sector for Evidence-Based Healthcare, National Institutes of Health, Ministry of Health, Shah Alam 40170, Malaysia; 4 Department of Ophthalmology, Kuala Lumpur Hospital, Kuala Lumpur 50586, Malaysia; 5 Department of Ophthalmology, Selayang Hospital, Shah Alam 68100, Malaysia; 6 Office of Director General of Health, Ministry of Health, Putrajaya 62590, Malaysia; 7 National Institutes of Health, Ministry of Health, Shah Alam 40170, Malaysia

**Keywords:** MAIWP, Hospital Selayang, cataract, cataract surgical rate, age adjusted rate

## Abstract

Introduction. Cataract is the leading cause of blindness. About 90% of cataract blindness occurs in low- and middle-income countries. The prevalence of blindness and low vision in any country depends on the socioeconomic status, the availability of medical and healthcare facilities, and the literacy of the population. Aim: This paper aims to estimate the cataract surgery rate (CSR) at Pusat Pembedahan Katarak, MAIWP-Hospital Selayang (Cataract Operation Centre), and provide descriptive assessments of the patients who received eye treatments in the center. Methods: The data were retrieved from the clinical database from 2013 to 2016. Information on the patient’s sociodemographic and clinical and treatment history was collected. Results: The cataract surgery rate for 2013 was about 27 and increased to 37.3 in 2014. However, it declined to 25 in 2015 before it resumed to 36 in 2016. For female patients who received eye treatments at Pusat Pembedahan Katarak, MAIWP-Hospital Selayang, the rate was higher (53.7%) compared to male patients (46.3%). The mean duration of cataract surgery from 2013 to 2016 was 21.25 ± 11.071 min. Conclusion: The increased cataract surgery rate for MAIWP-HS through smart partnerships for day care cataract surgery proved that better accessibility makes the short- and long-term strategies for the reduction and prevention of blindness in Malaysia possible to achieve.

## 1. Introduction

The World Report on Vison (WRV), which was released by the World Health Organization (WHO), stated that 2.2 billion people across the world have a vision impairment. Of these, a minimum of 1 billion have a preventable vision impairment [1]. This report has suggested that the delivery of universal eye health coverage (UEHC) can be optimized through an integrated, people-centered eye care (IPCEC). Therefore, the identification and development of a national health delivery system is crucial to improve the quality and quantity to prevent and address vision impairment [2].

The majority (80%) of blindness can be prevented. There are many causes of blindness among adults and children. For adults, cataract has been identified as one of the leading causes of blindness and has affected more than 18 million people. About 95% of blindness from cataract occurs in low- and middle-income countries (LMICs) and accounts for 51% of the total cases of blindness [3]. Among children, blindness can be due to measles and a vitamin A deficiency [4].

The major causes of blindness vary widely across the continents, which is related to the level of socio-economic development across the countries, the availability of medical and healthcare facilities and the literacy of the population.

In Malaysia, the most common causes of blindness and low vision are cataract, refractive errors, glaucoma, diabetic retinopathy and retinopathy of prematurity [5]. A survey conducted by the University of Malaya Medical Centre among the urban population in Kuala Lumpur Federal Territory in 2014 [6] and Klang Valley in 2008 revealed the prevalence of cataract at 32.9%, glaucoma as 23.4% and diabetic retinopathy at 9.6% [7].

The Malaysia National Eye Survey conducted in 2014 found that age and gender adjusted the prevalence of blindness, severe visual impairment and moderate visual impairment, by 1.2%, 1.0% and 5.9%, respectively. The most common causes of blindness were untreated cataract (58.6%), diabetic retinopathy (10.4%) and glaucoma (6.6%). The majority of the causes of blindness (86.3%) were avoidable, and 58.6% of the causes of blindness were treatable [8].

Lens extraction surgery is the only effective method to treat cataract and is the most commonly performed ocular procedure in the world. With the increase in safety and improvements in visual outcomes, cataract surgery using intraocular lens implantation is now increasingly performed for the treatment of other conditions, including refractive error and angle closure glaucoma [9,10,11]. Cataract surgery has evolved from manual cataract extraction to phacoemulsification using an ultrasonic technique and laser-assisted cataract surgery that brings more precise incision, fewer complications and a better visual outcome. Therefore, cataract surgery requires a greater cost, highly trained people to perform the surgery and longer learning curves [12]. Presently, cataract surgery is available in both public and private hospitals in Malaysia.

The Federal Territory Islamic Religious Council, officially named the Majlis Agama Islam Wilayah Persekutuan (MAIWP), was established on 1 February 1974 under the Prime Minister Department, simultaneously with the establishment of the Federal Territory of Kuala Lumpur, Malaysia, which is situated in Klang Valley, the suburban area between Selangor and the Federal Territory. The establishment of this council aims to take care of Islamic affairs in the Federal Territory of Kuala Lumpur, which was previously placed under the Selangor state government. The MAIWP is responsible for managing the affairs of Muslims in Labuan and Putrajaya after they were placed under Federal Territories on 16 April 1984 and 1 February 2001, respectively.

One of the strategies of the MAIWP is to strengthen the efforts to improve the welfare of the people residing within the Federal Territories and increase the assets belonging to the council through investments and other halal ventures for the benefit of the people. Pusat Pembedahan Katarak, MAIWP-Hospital Selayang (Cataract Operation Centre, MAIWP-Hospital Selayang) which started operations on 16 January 2013, is a dedicated center for cataract surgery and is the only successful center in Southeast Asia that was developed from a strategic initiative and inter-agency collaboration between the MAIWP and the Ministry of Health (MOH), Malaysia, through the Selayang Hospital, with the aim to help underprivileged patients get medical attention and treatment. This initiative is a unique and dynamic national strategy platform that brings together ministries, agencies, all levels of government and the private sectors on a voluntary basis. The design and selection of this initiative are based on two key principles, namely, delivering a high income through economic growth and integrated development and enhancing the level of public well-being through greater security as well as social inclusion. Therefore, this initiative will be able to close the social distance between various groups in Malaysian society, for example, rural vs. urban, young vs. old, or men vs. women [13]. Subsequently, the MAIWP distributed almost MYR 10 million to renovate their building premises into a surgical center equipped with sophisticated and up-to-date surgical equipment.

**Figure 1:**
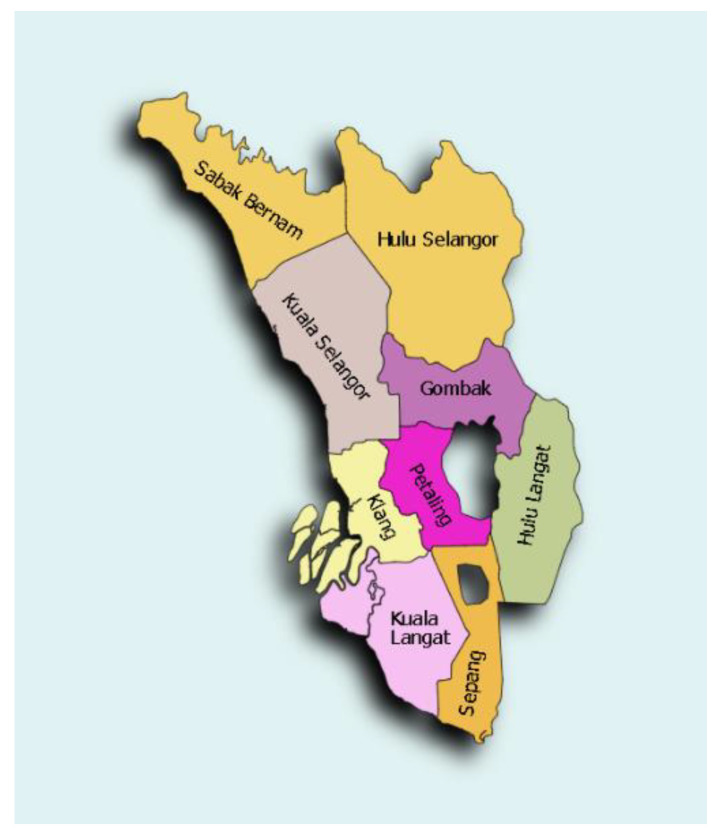
Map of Selangor showing the nine districts encircling Federal Territory, Kuala Lumpur.

The Selayang Hospital is situated in the suburbs of the Gombak district in Selangor, with a population of almost one million [14]. Selangor is a state on the west coast of Peninsular Malaysia with almost seven million people in the total population, encircling Kuala Lumpur, the capital of Malaysia (Figure 1). It comprises of nine districts: Gombak, Klang, Kuala Langat, Kuala Selangor, Petaling, Sabak Bernam, Sepang, Hulu Langat and Hulu Selangor. There are four major cities in Selangor, namely, Shah Alam, Klang, Kajang, Petaling and Subang Jaya, which are defined as urban areas, while the other districts are in the suburbs.

There are many public hospitals that perform cataract surgery in Selangor, including the Selayang Hospital, Shah Alam Hospital, Klang Hospital, Sungai Buloh Hospital and Serdang Hospital. However, Selayang Hospital is the only primary referral center with four other subspecialties in ophthalmology. The hospital received a high number of patients to undergo cataract surgery. It is a public hospital funded by the Malaysian government. Therefore, all resources, including human resources, salaries and other expenditures, are covered by the government. The doctors receive training through various master’s programs at public or private universities, locally or internationally, before being awarded with a master’s degree in their respective fields. They have to undergo an extensive one-year training and subspecialty training according to their chosen fields after the master’s program. A master’s in ophthalmology is one of the training programs for the doctors who are interested in pursuing a career in this area. They will be given continuous training during the master’s program and will be assigned to the specialists expert in the field.

Due to the lack of space and the high number of patients received in Selayang Hospital, cataract patients have to wait up to a year before surgery can be performed. This hospital has two fully functioning operating theatres, which includes all other cataract care pathways under one roof. The ophthalmology clinic and other surgical clinics sessions open daily from Monday to Friday. Therefore, all the scheduled routine cataract surgeries are performed in Pusat Pembedahan Katarak, MAIWP by the ophthalmologists from Selayang Hospital to shorten the waiting period and increase the cataract surgery rates with high-quality service and good outcomes among all the scheduled cataract surgery cases. Cataract surgery is funded by MAIWP, whereas Selayang Hospital provides the service, including human resources and medical services, including the follow up after surgery [15].

If the incidence of cataract is higher than the total number of cataract surgeries, this will lead to an increased backlog of people who require cataract surgery. With the establishment of the Pusat Pembedahan Katarak, MAIWP-Hospital Selayang, the waiting period for cataract patients to undergo surgery has been reduced from sixteen to two weeks.

## 2. Materials and Methods

### 2.1. Ethical Approval

This study was registered under the Malaysia National Medical Research Registry (NMRR) with the identification number NMRR-19-3151-51710 and was funded by the MOH operational budget. The MOH, Malaysia, allows the use of secondary data from either registry or hospital clinical databases, provided that the data are anonymized. Therefore, the data were de-identified prior to analysis.

### 2.2. Data Collection

Since 2013, with the implementation of government policies on smart partnership, data for all cataract patients who had operations in Hospital Selayang were recorded in a clinical database using a standardized information form called a “Registration Form”, which contains information on the following: patient’s name, gender, age, ethnicity, home address, cataract classification, detailed ocular examination, such as eyelid anatomy and inflammation, presence of abnormalities in the cornea, fundal examination for retina abnormalities, biometry test, naked eyesight, visual acuity, intra-ocular pressure, surgeon’s name, surgical management, whether they have had intraocular lens (IOL) implantation or not and operation outcome (mainly 1st–3rd postoperative day corrected visual acuity (VA)). All surgeons who performed the cataract surgeries must fill out the registration form by the 10th day of the following month.

For this study, records were retrieved from the clinical database from Hospital Selayang, Selangor, Malaysia. A total of 2266 patients underwent cataract surgery at Pusat Pembedahan Katarak, MAIWP-Hospital Selayang from 2013 to 2016. All the patients’ information was retrieved from the clinical database. Data from all cataract patients were extracted from the data registration form. The information from the registration forms were transferred to the local Eye Clinic Management System (ECMS), which synchronizes with National Eye Database (NED) at regular intervals. NED is a database supported by MOH. It is an eye health information system that contains a clinical database consisting of six patient registries and a monthly ophthalmology service census. The patient registries are Cataract Surgery Registry, Diabetic Eye Registry, Contact Lens-Related Corneal Ulcer Surveillance, Glaucoma Registry, Retinoblastoma Registry, Retinoblastoma Registry and Age-Related Macular Degeneration Registry.

### 2.3. Definition

This paper used the WHO definition of blindness, low vision and visual impairment. Blindness was defined as presenting a visual acuity of less than 3/60 or as the inability to count fingers at a distance of three meters in the better eye using the available means of correction (with spectacles when available). Low vision was defined as presenting a visual acuity of less than 6/18 but with an equal or greater score of 3/60 in the better eye when using the available means of correction (with spectacles when available). Visual impairment was defined as presenting a visual acuity of less than 6/18 in the better eye when using the available means of correction (with spectacles when available).

Cataract was defined as the presence of lens opacity, with a grey or white appearance of the pupil when examined with an oblique light in a shaded or darkened area. Refractive errors were defined as visual impairment that improved to 6/18 or better with a pinhole, with no evidence of cataract in a torchlight examination. Retinal diseases were defined as retinal abnormalities caused by dystrophy, degeneration or acquired metabolic causes, such as diabetes mellitus. Glaucoma was defined as the presence of the horizontal cup-disc ratio of 0.4 or more along with an intraocular pressure of more than 22 mm Hg. Corneal diseases were defined as loss of normal corneal transparency due to whatever causes involving the central cornea.

The cataract surgery rate (CSR) was defined as the number of cataract surgeries per million people per year, and it is a critical index to demonstrate that cataract blindness is becoming eliminated.

### 2.4. Data Analysis

An analysis was performed using the Statistical Package for Social Science, version 26.0 (SPSS, Inc., Chicago, IL, USA) for Windows. The patients’ characteristics were summarized for the entire sample using the mean values and standard deviations (SDs) for continuous variables and frequencies and percentages for categorical variables.

## 3. Results

In tandem with the MOH strategy to address the issue of cataract blindness in this country, 1576 patients underwent cataract surgery in 2013, which increased to 2255 in 2014. However, the number decreased to 1543 in 2015. A slight increase can be seen between 2013 and 2016, with the number of cases in 2016 amounting to 2266. The mean age for cataract patients in 2013 to 2016 was 64.57 ± 8.436 years old. The majority (70%) of patients who went for cataract surgery were 60 years and above, each year between 2013 to 2016. Patients below 45 years old contribute cumulatively to about 1% of all the cataract surgeries performed between 2013 and 2016. Overall, the mean duration of a cataract surgery for each patient was 21.25 ± 11.071 min between 2013 to 2016.

In term of gender, more than 50% of the patients were female in all four years of this study. Patients were seen on a day care basis, and the majority (95%) went for the phacoemulsification technique. From 2013 to 2016, about 42.2% of the patients who received treatment were Malay, 39.7% were Chinese, 15.4% were Indian and 2.7% were of other races. Almost 100% of the patient went for IOL implantation instead of another choice (Table 1).

At the beginning of the partnership, the CSR for 2013 was 26.69, as was observed in 2015. However, in 2016, the CSR increased to 36.02 (Table 2). The percentage of phacoemulsification surgeries performed in the center increased from 95% to almost 100%. From 2013 to 2016, the IOL implantation rate was greater than 98%, and more than 75% of patients had a 1st–3rd postoperative day corrected VA ≥ 0.3.

## 4. Discussion

The increment in cataract surgery rates from 2013 to 2016 for the MAIWP-HS indicated a progress and demonstrates the increasing availability of eye healthcare in Malaysia. CSR is a critical index to demonstrate that cataract blindness is being eliminated. The rate is higher in well-developed and high-income countries. For example, Japan recorded 10,198 cases per million people in 2013, and Australia recorded 7202 cases per million people in 2014. However, the rate is still very low in some parts of Asia. For example, China recorded 1402 cases per million people in 2015, and Indonesia recorded 1411 cases per million people in 2014 [16,17].

In Malaysia, the CSR is still low. For example, in 2014, 1397 cases per million people were recorded [17]. There are many factors associated with the CSR, such as the awareness, prevalence of cataract, accessibility to health facilities, the racial and genetic make-up and the density of ophthalmologists. However, a country with a high economic level but a low prevalence of cataract is unlikely to produce a high CSR [18,19,20]. The most common factors for a poor uptake of cataract surgery were ‘awareness’ (43.5%) and ‘fear of surgery or poor outcome’ (16.2%), respectively [17]. A study conducted by Lee MY et al., showed that the number of cataract surgeries performed across Malaysia as a day care service increased from 4887 (39.3%) in 2002 to 14,842 (52.3%) in 2011. This trend may be due to the improvement of the surgical capacity in government hospitals and the change in practice patterns among ophthalmologists [12].

Our results also showed that female patients constituted more than 50% of all cataract surgery patients each year, and the age-adjusted rate of cataract surgery (per 100,000 inhabitants) has been significantly higher for females than for males in the age group between 60 and 79 years old. The differences in gender may be due to a higher prevalence of cataract in female subjects [21]. This finding is similar to a study conducted in China, which showed that females had a significantly higher prevalence of visual impairment than men [22]. The risk of cataract increases with age [23]. Therefore, as women have a longer life expectancy than men, this may contribute to their high cataract burden [24]. According to the study by Resnikoff et al., women are more likely to have a visual impairment compared to men in every region of the world [25]. Olofsson, et al. found that female subjects who had cataract surgery also visited the healthcare provider for other reasons more frequently than the male subjects [26]. This may also contribute to the higher rate of surgery for female subjects. Additionally, a study suggested that estrogen may play a role in the incidence of cataract among women. A decrease in estrogen during menopause may cause an increased risk of cataract in women. However, it may not be due the concentration of the hormone, but to the withdrawal effect [27].

Phacoemulsification is the preferred technique for cataract surgery in Malaysia. It has increased significantly from 39.7% in 2002 to 78.0% in 2011. On the other hand, extracapsular cataract extraction (ECCE) has dropped from 54.0% in 2002 to 17.3% in 2011. Phacoemulsification contributed toward about two thirds of the total cataract surgeries completed at the MOH hospitals in 2011. This may be due to the financial cost, as a significant number of hospitals did not have phacoemulsification machines in the early 2000s (12). Studies by Thevi, Reddy and Shantakumar in Pahang and Soundarajan et al. in Malaysia concluded that the visual outcome was significantly better with phacoemulsification compared to the ECCE procedure (*p* = 0.001), and they recommended that phacoemulsification equipment should be supplied to the district hospitals with adequate facilities, to assist with the performance of intraocular surgery [6,28].

According to Yorston, apart from the cost of surgery and IOL, the lack of awareness, poor service and long distances from surgical centers were the largest hindrances of cataract surgery in developing countries [29]. A study conducted in Shanghai showed that many patients in suburban areas were not willing to have cataract surgery, even though the cataract surgery services were easily available, and the authors suspected that it may be due to the lack of awareness [30]. Therefore, a program must be developed to raise awareness in the suburban population, to improve people’s knowledge, understanding and willingness to accept cataract surgery as an intervention to reduce reversible blindness. Increased rates of cataract surgeries in private hospitals in recent years may also contribute to the CSR in government facilities. Patients are more willing to pay for cataract surgery despite the surgery’s actual cost, due to the high-quality service, accessibility and convenience [31]. The healthcare service in Malaysia is divided into two highly developed sectors. The main provider is the MOH, which is funded through the general taxation of income, and the second provider is via the private sector, which has grown substantially over the last 30 years. Public healthcare provides a service to the majority (65%) of the population but is served by just 45% of all registered doctors, and even fewer specialists. Therefore, patients only need to pay minimum fees in the heavily subsidized public sector. However, the nominal fees in this public system are only applicable for Malaysian nationals. Foreigners are eligible for public healthcare, or they can choose a private healthcare facility with additional fees. Private healthcare offers a similar service to public healthcare at a higher cost, but the services are much faster and more comfortable because of the higher numbers of doctors and hospitals in this sector. However, the quality of the staff or equipment between the public and private sectors is similar [32].

The cases of blindness caused by cataract are increasing globally by approximately one million per year, and the cases of reversible blindness caused by cataract with a visual acuity of less than 6/60 are increasing by 4–5 million per year. Therefore, to reduce the backlog of cataract cases, it is necessary to increase the uptake of cataract surgery. It is possible to achieve these rates if high-quality cataract surgery is performed at a reasonable cost and close to where people live, using the cataract operation center, MAIWP-HS, as a model. This model, as an outreach center, has now been developed in several countries, most prominently in India [33,34].

The CSR can be increased by implementing a better policy for patients from poor economic backgrounds. Thus, it is important to identify the income source for patients to enable them to receive the service at a minimal cost. Other provisions include transportation to pick up patients, drive them to the health facility, perform the surgery and then send them back home afterwards. In these situations, more patients who have low incomes and difficulty commuting will accept cataract surgeries. This will ensure the delivery of high volume, good quality and low-cost cataract surgical services. Advocating with colleagues in other health sectors and with governments and private healthcare services will maximize the delivery of the essential eye care for those from marginalized groups and will achieve the goal of Vision 2020 [35].

There are a few limitations in this study. One of the limitations is that the data for the long-term follow-up of cataract surgery patients is impossible to retrieve, due to improper record keeping. Another limitation is that the outcome of cataract surgery was only measured from the 1st–3rd postoperative day on the corrected VA, without the long-term post-operative outcome of cataract surgery. The CSR data does not incorporate other significant outcomes, such as the sight restoration rate or the cataract surgical coverage among the cataract blind. Furthermore, the CSR data, prior to the setting of the center, were not available for Selangor.

## 5. Conclusions

The increase in the cataract surgery rate from 2013 to 2016 for the MAIWP-HS as a reference center for day care cataract surgery is a start, and it is going to help decision-makers achieve the short- and long-term strategies for the reduction and prevention of blindness in Malaysia. More awareness programs on cataract surgery in the suburban population are needed to improve knowledge and awareness with regards to cataract and its complications.

## Figures and Tables

**Table 1:** Basic characteristics of the patients registered for cataract surgery in MAIWP-HS, from 2013 to 2016.

		Year				Total
		2013	2014	2015	2016	
Pre-clerking conducted at	Hospital Klang	0	0	5	0	5
	Hospital Selayang	25	106	9	295	435
	Hospital Serdang	48	46	17	0	111
	Hospital Sungai Buloh	4	0	0	0	4
	MAIWP-Hospital Selayang	1499	2103	1512	1971	7085
Operative conducted at	MAIWP-Hospital Selayang	1576	2255	1543	2266	7640
Age group	0–4	0	1	0	0	1
	15–19	2	0	0	0	2
	20–24	2	1	0	1	4
	25–29	1	0	0	3	4
	30–34	0	4	1	2	7
	35–39	5	4	8	9	26
	40–44	18	31	16	18	83
	45–49	48	68	50	62	228
	50–54	120	155	110	157	542
	55–59	204	329	232	270	1035
	60–64	322	448	314	533	1617
	65–69	349	529	388	597	1863
	70–74	324	437	260	370	1391
	75–79	142	213	155	199	709
	80–84	35	30	9	43	117
	85 and above	4	5	0	2	11
Gender	Male	719	1073	718	1030	3540
	Female	857	1182	825	1236	4100
Ethnic Group, other specify	Malay	613	984	684	940	3221
	Chinese	705	843	602	884	3034
	Indian	225	378	204	371	1178
	Others	33	50	53	71	207
Surgery on	First eye	891	1272	912	1391	4466
	Second eye	608	846	599	829	2882
	Not available	77	137	32	46	292

**Table 2:** Analysis of Cataract Surgeries in Cataract Operation Centre, MAIWP-Hospital Selayang 2013–2016.

Year	No CS	Population in Selangor (Million)	CSR	Surgical Method (%)			Snellen Visual Acuity	
							A	B
				ECCE	Phaco	Others	≥60/120 (%)	≥6/19 (%)
2013	1576	5.904	26.69	4.2	95.0	0.8	99.6	75.6
2014	2255	6.051	37.27	2.5	97.0	0.5	99.7	80.0
2015	1543	6.178	24.98	1.6	98.1	0.3	99.6	84.6
2016	2266	6.291	36.02	1.1	98.2	0.7	99.6	81.6

Abbreviations used: No. CS—the number of cataract surgeries; Phaco—phacoemulsification surgeries; ECCE—extracapsular cataract extraction surgeries; A%—percentage of patients with 1st–3rd postoperative day corrected Snellen visual acuity (VA) ≥ 60/120; B%—percentage of patients with 1st–3rd postoperative day corrected Snellen VA ≥ 6/19.

## Data Availability

The data that support the findings of this study are available upon request from the corresponding author, NAM. The data are not publicly available due to information that could compromise the privacy of patients.
